# Tang-Tong-Fang Confers Protection against Experimental Diabetic Peripheral Neuropathy by Reducing Inflammation

**DOI:** 10.1155/2015/574169

**Published:** 2015-10-11

**Authors:** Mingdi Li, Da Huang, Xiaoxing Liu, Lan Lin

**Affiliations:** Guang'anmen Hospital, Chinese Academy of Chinese Medical Sciences, Beijing 100053, China

## Abstract

Tang-tong-fang (TTF) is a Chinese herbal formula that has been shown to be beneficial in diabetic peripheral neuropathy (DPN), a common complication secondary to diabetic microvascular injury. However, the underlying mechanism of protection in nerve ischemia provided by TTF is still unclear. We hypothesized that TTF alleviates DPN via inhibition of ICAM-1 expression. Therefore, we tested the effect of TTF in a previously established DPN model, in which nerve injury was induced by ischemia/reperfusion in streptozotocin-induced diabetic rats. We found that the conduction velocity and amplitude of action potentials of sciatic nerve conduction were reduced in the DPN model group but were rescued by TTF treatment. In addition, TTF treatment also attenuated the effect of DPN on other parameters including histology and ultrastructural changes, expression of ICAM-1, MPO, and TNF-*α* in rat sciatic nerves, and plasma sICAM-1 and MPO levels. Together, our data suggest that TTF treatment may alleviate DPN via ICAM-1 inhibition.

## 1. Introduction

Diabetes mellitus is one of the most morbid diseases worldwide and, according to projections by the World Health Organization, the adult diabetic population is expected to grow by 170% between 1995 and 2025 [1]. Diabetic peripheral neuropathy (DPN) is the most common complication of diabetes, affecting almost half of patients with diabetes [2]. A myriad of therapeutic modalities, including several currently in clinical trials, have been used to treat DPN with limited efficacy. The only known prevention strategy for DPN remains tight glucose control [3].

Diabetic medical experts have recently turned their attention towards complementary and alternative medicine as possible avenues to identify new therapeutic strategies for diabetic complications [4]. Specifically, it has been reported that traditional Chinese medicine or natural medicine has had some success in the treatment of DPN. Existing studies focus on nerve repair and regeneration, Schwann cell survival signaling pathways, neurotrophic factor, free radicals, NF-*κ*B, TNF-*α*, and IL-1 [4–9]. However, though previous studies suggest that plasma cell adhesion molecules (CAMs) play an important role in the development and progression of peripheral neuropathy in diabetes mellitus, the role of adhesion factor in nerve microvasculature during the development of neuropathy has not been explored.

Cross-sectional studies have shown plasma cell adhesion molecules to be increased in patients with diabetic complications [10]. Plasma CAM expression may even predict the development of diabetic neuropathy. Specifically, intercellular adhesion molecule-1 (ICAM-1) is an important CAM produced by vascular endothelial cells, which mediates the interaction of polymorphonuclear neutrophils (PMN) with vascular endothelial cells and may serve as an independent factor in the pathogenesis and progression of DPN [10]. ICAM-1 has an important role in the regulation of neutrophil adhesion to the endothelium and capillary wall permeability [11]. Under normal conditions, the expression of ICAM-1 is low or absent. However, recent studies have shown that the expression of ICAM-1 in the nerve tissue microvasculature of ischemia-reperfusion DPN model rats is increased and aggravates axonal degeneration [12]. A previous study has demonstrated that the circulating levels of ICAM-1 are elevated in DPN rats [13]. Blocking intracellular adhesion molecule-1 (ICAM-1) prevents leukostasis and retinal vascular leakage [14].

Tang-tong-fang (TTF) is a Chinese herbal formula developed by Professor Lin from Guang'anmen Hospital. The main ingredients of TTF consist of* Astragalus*,* Ligusticum wallichii*, Ramulus Cinnamomi, Radix Paeoniae Alba,* Eupolyphaga*,* Curcuma*, and Asiasarum. In traditional Chinese medicine, this formula has been used to improve circulation. Clinically, TTF has been shown to be effective in the treatment of DPN [15]. Past clinical trials have confirmed that TTF can increase nerve conduction velocity and ameliorate lower extremity sensory changes [15]. However, no research exists exploring the mechanism of the therapeutic benefit provided by TTF.

We, therefore, hypothesized that TTF may improve DPN-related nerve injury through the inhibition of ICAM-1 expression. We examined nerve conduction velocity of DPN rats as well as histopathology and ultrastructural changes of sciatic nerve tissue. In order to further characterize the mechanism of TTF in improving DPN, we also measured the expression of ICAM-1, myeloperoxidase (MPO), and TNF-*α* in sciatic nerve tissue, as well as plasma levels of ICAM-1 and MPO.

## 2. Materials and Methods

### 2.1. Animals

One hundred and ten male Sprague-Dawley (SD) rats (body weight = 180 ± 10 g) (Beijing HFK bioscience Co. Ltd., license number: SYXK (E) 2009-0007, Beijing, China) were randomly divided into a control and a diabetic model group. Controls (*n* = 18) were injected with citrate buffer alone. In the model group (*n* = 92), diabetes was induced by intraperitoneal injection of STZ in 0.1 mol/L citrate buffer at pH 4.2 (20 mg/mL; dose 60 mg/kg). Successful induction of diabetes was confirmed when fasting blood glucose exceeded 16.7 mmol/L three days after injection of STZ and remained >16.7 mmol/L throughout the study. All of the following experiments conformed to the Guiding Principles for the Care and Use of Laboratory Animals issued by the National Committee of Science and Technology of China.

### 2.2. Drugs and Solutions

TTF was provided by the Guang'anmen Hospital. The medicine was boiled, vacuum-packaged, preserved under 4°C, and diluted before use, according to standard protocols.

### 2.3. Creation of the DPN Model

The DPN model was established by inducing ischemia-reperfusion (IR) in diabetic animals as previously described [16, 17]. After a four-week induction period, the STZ-diabetic rats were anesthetized with intraperitoneal pentobarbital. Ischemia was produced by occlusion of the abdominal aorta, right common iliac, and femoral artery with artery clips, which were removed after three hours. Sixty-seven rats were included in the final study, as twenty-five rats were excluded either for death during surgery (*n* = 18) or due to an insufficient increase in fasting blood glucose (<16.7 mmol/L) (*n* = 7).

### 2.4. Animal Groups and Treatment

The sixty-seven IR rats were initially divided into four groups: model (*n* = 18), low-dose TTF (*n* = 16), medium-dose TTF (*n* = 17), and high-dose TTF (*n* = 16). Respectively, the three TTF groups were dosed with TTF 3.5 times (5.15 g/kg/d), 7 times (10.30 g/kg/d), or 14 times (20.60 g/kg/d) for eight weeks. The control group and model groups were treated with distilled water (10 mL/kg/day) for eight weeks.

### 2.5. Plasma Biochemical Parameters Measurement

Plasma soluble ICAM-1 (sICAM-1) was measured by the STAT FAX 2100 automatic enzyme immunoassay instrument (Awareness Technology Inc., USA) using rat sICAM-1/CD54 ELISA Kit (R&D Systems Inc., USA). Plasma MPO was measured by UV-2000 UV Spectrophotometry (Unico Instrument Co. Ltd., China) using a commercially available MPO Kit (Nanjing Jiancheng Bioengineering Institute, China).

### 2.6. Electrophysiology

The conduction velocity (CV) and amplitude of nerve action potentials (NAP) were measured by the BL-420F biological function experimental system (ChengDu Technology & Market Co. Ltd., China) using stimulating and recording electrodes. After anesthesia with intraperitoneal pentobarbital, the right sciatic nerves were exposed by blunt dissection. The nerve was stimulated proximally and the electric signal recorded from the distal digital nerve. The CV of tails were measured in a similar manner with single needle stimulating electrodes inserted into the proximal tail and recording electrodes inserted distally. The time (*T*) and distance (*S*) between the stimulating electrodes and recording electrodes were used to calculate CV using the equation CV = *S*/*T*.

### 2.7. Neuropathology

Following electrophysiological testing, the right sciatic nerves were excised and portions fixed with 3% glutaraldehyde for one week, followed by 1% osmium tetroxide fixation for 1.5 hours and dehydration in a step-wise manner with 50%–100% alcohol, soaked in epoxy resin and acetone mixture for two hours, embedded in epoxy resin, and then cut into semithin sections (1–3 *μ*m) and ultrathin sections (50–70 nm) by an EM UC7 ultrathin slicing machine (Leica Ltd., Germany). The semithin sections were stained with 1% toluidine blue and observed with a DM-3000 biological microscope (Leica Ltd., Germany) under 200x magnification. The ultrathin sections were stained with uranium-lead staining and observed with a JEM-1400 transmission electron microscope (JEOL Ltd., Japan) under 8000x magnification.

### 2.8. Immunohistochemistry

Portions of right sciatic nerve were also fixed by 10% formalin phosphate buffer, dehydrated by alcohol, infiltrated and embedded in liquid paraffin, and cut into paraffin sections by a RM2255 rotary slicing machine (Leica Ltd., Germany). The following reagents were used: ICAM-1 antibody (Santa Cruz, USA), anti-TNF alpha antibody (Abcam, Britain), and ultrasensitive two-step immunohistochemical detection reagent (OriGene-Your Gene Company, China). Paraffin sections were dewaxed, incubated in 3% H_2_O_2_ deionized water for ten minutes, repaired by microwave antigen, incubated in ICAM-1/Anti-TNF alpha antibody overnight at 4°C and ultrasensitive two-step immunohistochemical detection reagent for twenty minutes at room temperature, stained with diaminobenzidine (DAB), and then examined by microscope with 200x magnification. The integral optical densities (IOD) were measured using Image-Pro Plus 6.0 software.

### 2.9. Western Blot Analysis

Portions of the right sciatic nerves were also immediately harvested for Western blot analyses. The following reagents were used: ICAM-1 antibody (1 : 300, Santa Cruz, USA), MPO heavy chain antibody (1 : 300, Santa Cruz, USA), Beta actin antibody (1 : 1000 OriGene-Your Gene Company, China), goat anti-mouse IgG (H + L), HRP (1 : 10000 Jackson ImmunoResearch Laboratories Inc.), BCA Protein Assay Kit (CW Biotech, China), and PVDF membrane (Merck Millipore, China). Western blot analyses were performed using standard methods. Briefly, the tissues were homogenized by RIPA buffer, and extracted proteins were separated by 10% SDS-PAGE and transferred onto PVDF membranes, which were then incubated with the indicated antibodies overnight at 4°C. The membranes were further incubated with horseradish peroxidase-conjugated goat anti-mouse IgG for one hour at room temperature. Bands were visualized by chemiluminescence.

### 2.10. Real-Time PCR Detection

Total RNA was extracted from fresh-frozen neural tissue using an Ultrapure RNA Kit (CWbio Co. Ltd., China) and then reverse-transcribed with a HiFi-MMLV cDNA Kit (CWbio Co. Ltd., China). Real time PCR was performed on a Bioer line gene PCR instrument (BIOER, China) using Invitrogen primers. The PCR amplification procedure includes 95°C for 10 min, followed by 45 cycles of 95°C for 15 s and 59°C for 60 s. Data were normalized to Beta actin.

### 2.11. Statistical Analysis

Statistics were performed using SPSS 18.0. Significant differences between means were calculated by one-way analysis of variance (ANOVA), followed by multiple comparisons with least significant difference (LSD) test (equal variances) or Dunnett T3 test (unequal variances). Statistical significance was defined by *p* < 0.05.

## 3. Results

### 3.1. Effects of TTF on the Nerve Conduction Velocity of DPN Rats

**Figure 1:**
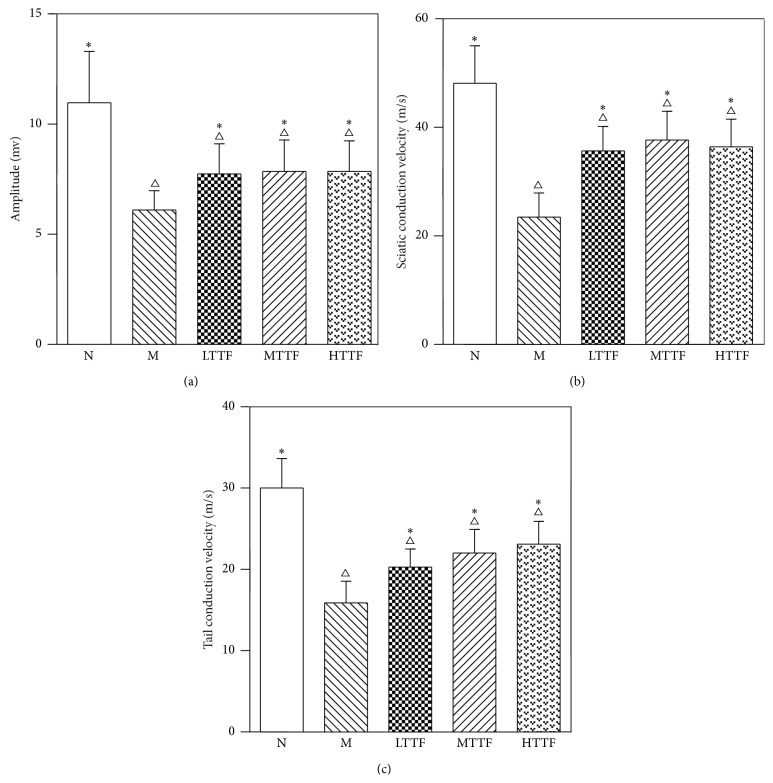
Action potential amplitude (a), sciatic nerve conduction velocity (b), and tail nerve conduction velocity (c) in N (control, *n* = 18), M (model, *n* = 16), LTTF (low-dose TTF, *n* = 16), MTTF (medium-dose TTF, *n* = 15), and HTTF (high-dose TTF, *n* = 15) groups (^∆^ *p* < 0.05 versus the control group and ^*∗*^ *p* < 0.05 versus the model group).

In order to determine the potential therapeutic effect of TTF on DPN, we first measured the effect of TTF on the nerve conduction velocity of DPN rats. Compared with the control (N) group, the sciatic nerve conduction velocity, action potential (AP) amplitude, and tail nerve conduction velocity were significantly reduced in the non-TTF diabetic model (M) group (N versus M; sciatic nerve conduction velocity (m/s): 48.12 ± 6.9 versus 23.47 ± 4.43; AP amplitude: 10.97 ± 2.33 versus 6.11 ± 0.88; tail nerve conduction velocity: 30.03 ± 3.63 versus 15.89 ± 2.67; *n* = 16–18, *p* < 0.01). Treatment with TTF at different doses was found to increase the sciatic nerve conduction velocity, AP amplitude, and tail nerve conduction velocity compared with those detected in the control group (*n* = 15-16, *p* < 0.01) (Figure 1).

### 3.2. Sciatic Light Microscopy Structure

**Figure 2:**
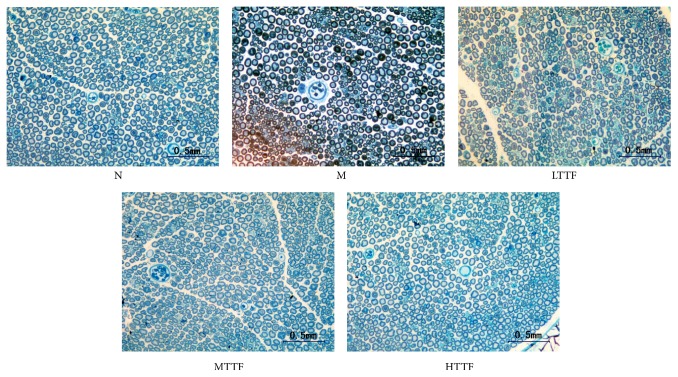
Sciatic light microscopy structure: semithin sections stained with toluidine blue. Control group (N): sciatic nerve fibers with full axons and even staining of myelin sheath. Model group (M): atrophied and misshapen axons with darkened and edematous myelin sheath. Low-dose TTF group (LTTF). Medium-dose TTF group (MTTF). High-dose TTF group (HTTF).

Since TTF treatment improves the nerve conduction velocity in DPN rats, we next hypothesized that TTF may protect the structure of nerve tissue against diabetic and ischemic insults. As shown in Figure 2, electron microscopy confirmed edematous and ischemic nerve myelin, atrophied and misshapen axons, and thrombus formation in capillaries in the DPN model group. Conversely, signs of clinical improvement in the TTF group were manifested by an observable decrease in abnormal nerve fibers.

### 3.3. Sciatic Nerve Ultrastructure

**Figure 3:**
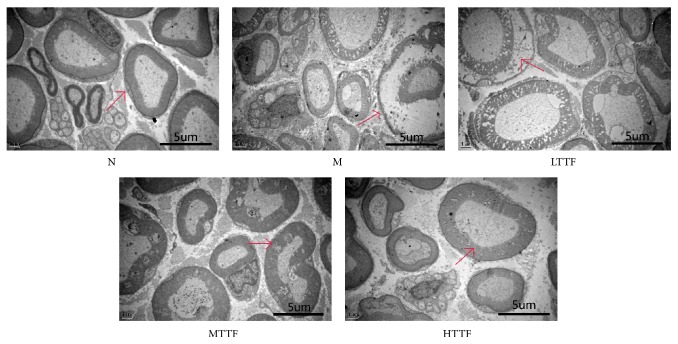
Sciatic nerve ultrastructure (uranium-lead staining). Control group (N): sciatic nerve with intact myelin sheaths, healthy axons with prolific cell organelles. Model group (M): the sciatic nerve with disrupted, tortuous myelin sheaths, axonal atrophy, and attenuation in cell organelles. LTTF: low-dose TTF group. MTTF: medium-dose TTF group. HTTH: high-dose TTF group.

Ultrastructural studies demonstrated edema in control sciatic nerves, as well as swelling and disruption of the myelin sheath, axonal atrophy, and reduction in cellular organelles. These observations are consistent with the observations obtained from light microscope. After TTF treatment, pathological changes were reduced in comparison to the control group, including mitigation of edema and injury to myelin sheath and axons (Figure 3).

### 3.4. Effect of TTF on Expression Level of sICAM-1 and MPO in Plasma of DPN Rats

**Figure 4:**
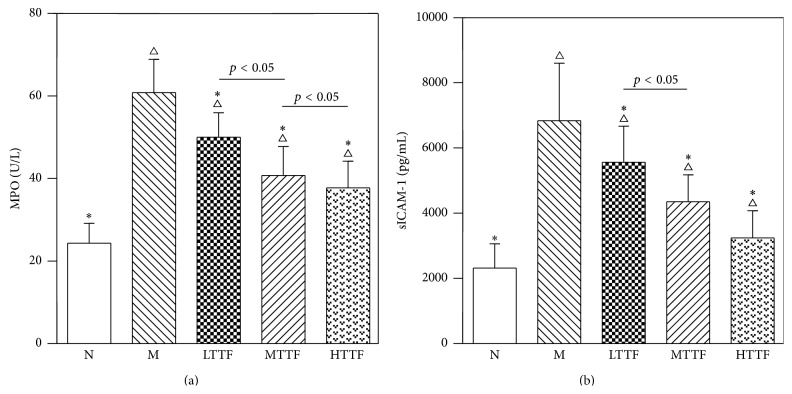
Expression level of MPO (a) and sICAM-1 (b) in plasma from N (control, *n* = 17), M (model, *n* = 15), LTTF (low-dose TTF, *n* = 16), MTTF (medium-dose TTF, *n* = 12), and HTTF (high-dose TTF, *n* = 15) groups (^∆^ *p* < 0.05 versus the control group, ^*∗*^ *p* < 0.05 versus the model group).

It was reported that sICAM-1 play an important role in the pathogenesis and progression of DPN [10]; whereas MPO is associated with various types of inflammation, its role in DPN has not been elucidated yet. Therefore, we measured the effect of TTF on expression levels of sICAM-1 and MPO in plasma of DPN rats. Compared with the control group, the levels of sICAM-1 and MPO in the model group were significantly increased (*p* < 0.01). After treatment with TTF, sICAM-1 and MPO levels in the TTF groups were reduced significantly compared with those in the model group (*p* < 0.01) in a dose-dependent manner (*p* < 0.05) (Figure 4).

### 3.5. Immunohistochemical Staining

**Figure 5:**
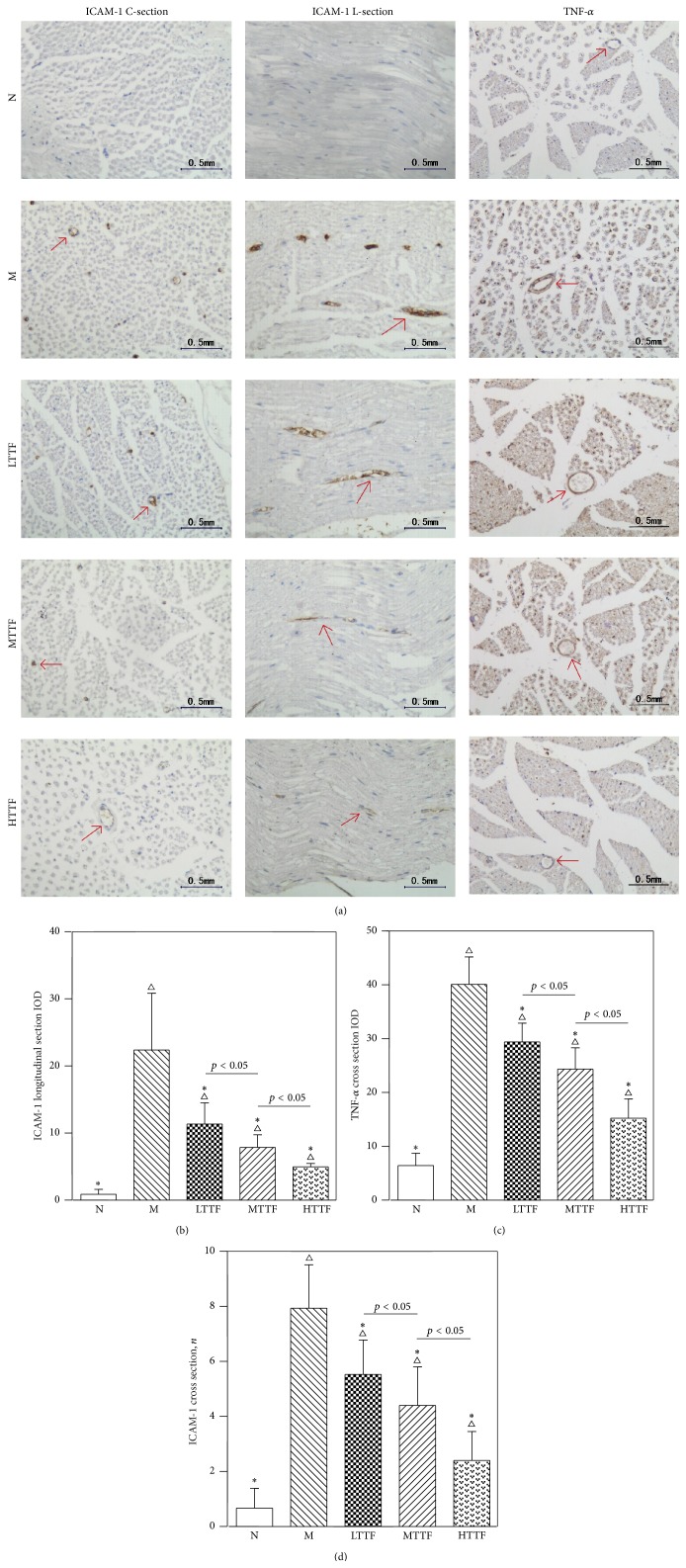
Immunohistochemical staining of ICAM-1/TNF-*α* in sciatic nerve. (a) Paraffin sections stained with ICAM-1/anti-TNF-*α* antibody. (b) The integral optical densities (IOD) of ICAM-1 staining in longitudinal section (*n* = 15 for each group). (c) The integral optical densities (IOD) of the TNF-*α* staining in cross section (*n* = 15 for each group). (d) The number of stained vessels by ICAM-1 in cross section (*n* = 15 for each group) (^∆^ *p* < 0.05 versus the control group, ^*∗*^ *p* < 0.05 versus the model group).

ICAM-1 and TNF-*α* have been demonstrated to play a major role in the nerve inflammation associated with DPN [10, 18]. Therefore, we measured the expression of ICAM-1 and TNF-*α* in sciatic nerves using immunohistochemical staining. Compared with the control group, the expression levels of ICAM-1 and TNF-*α* in sciatic nerves from the diabetic model group were significantly increased (*p* < 0.05). After treatment with different doses of TTF, expression levels of ICAM-1 and TNF-*α* in sciatic nerve was decreased significantly in the TTF groups compared with those in the model group (*p* < 0.05), with a demonstrated dose-dependent change (*p* < 0.05) (Figure 5).

### 3.6. Sciatic rtPCR Test

**Figure 6:**
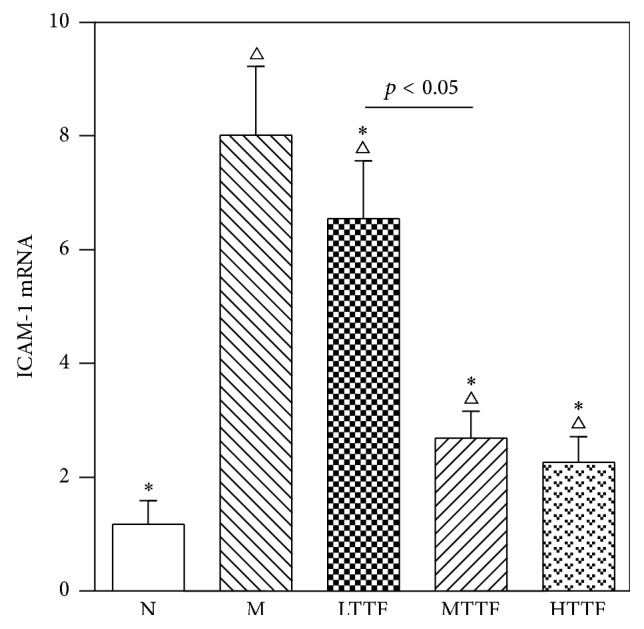
Real time PCR test of sciatic nerve from N (control, *n* = 5), M (diabetic, *n* = 5), LTTF (low-dose TTF, *n* = 5), MTTF (medium-dose TTF, *n* = 5), and HTTF (high-dose TTF, *n* = 5) groups (^∆^ *p* < 0.05 versus the control group, ^*∗*^ *p* < 0.05 versus the model group).

To confirm the observations obtained from immunohistochemical staining experiment. We further measured the mRNA expression of ICAM-1. Compared with the control group, the expression level of ICAM-1 mRNA in sciatic nerve in the model group was significantly increased (*p* < 0.01). After treatment with different doses of TTF, expression levels of ICAM-1 mRNA in sciatic nerve were significantly decreased in the TTF groups compared with the model group (*p* < 0.01) (Figure 6).

### 3.7. Western Blot of Sciatic Nerve

**Figure 7:**
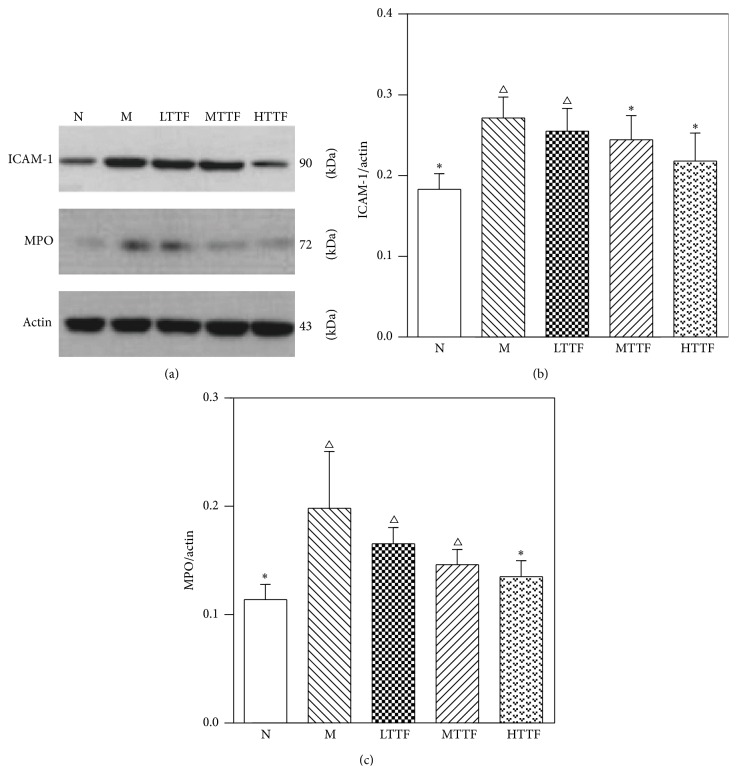
Western blot analysis of sciatic nerve. (a) Films of ICAM-1, MPO, and *β*-actin (control) bands. (b) Densitometric analysis of the ICAM-1 bands is expressed as integrated optical density (IOD), corrected for the corresponding *β*-actin (*n* = 5 for each group). (c) Densitometric analysis of the MPO bands (*n* = 5 for each group) (^∆^ *p* < 0.05 versus the control group, ^*∗*^ *p* < 0.05 versus the model group).

We further measured the protein expression of ICAM-1 and MPO in sciatic nerve to confirm the data obtained from previous experiments. Compared with the control group, expression levels of ICAM-1 and MPO in sciatic nerve from the diabetic model group were significantly increased (*n* = 5, *p* < 0.01). After treatment with different doses of TTF, the expression levels of ICAM-1 and MPO in sciatic nerve were significantly reduced in the TTF groups compared with the control group (*n* = 5, *p* < 0.01) (Figure 7).

## 4. Discussion

Diabetic peripheral neuropathy is characterized by symmetric sensory and motor dysfunction of peripheral nerves in distal extremities. DPN is one of the most serious comorbidities of diabetes and leads to significant morbidity and mortality due to sequelae such as ulceration, gangrene, and even limb loss. Diagnosis involves careful history and physical examination, as well as adjunctive studies including nerve conduction velocity and EMG examination [19].

The pathogenesis of DPN is multifactorial and is yet to be fully elucidated, though it is considered to be secondary to both hyperglycemia-induced pathologic changes intrinsic to neurons and ischemia-induced neuronal damage through decreased neurovascular blood flow [20, 21]. It is currently believed that this microvascular dysfunction is due to the reduction in nerve blood flow by alterations in vasoregulation of nerve microvessels and increased blood viscosity caused by hyperglycemia. Additionally, changes in the endothelium itself have been shown to involve AGE-RAGE-NF-*κ*B, cell adhesion molecules, nitric oxide, and peanut acid analogs [22].

Within the first week of development of diabetes, nerve blood flow is reduced by approximately 50% and hyperglycemia affects both sensory and sympathetic neuronal axons and cell bodies [23, 24]. Increased blood viscosity, increased platelet aggregation, and decreased red blood cell deformability together lead to decreased blood flow and tissue hypoxia [25]. Because of the vascular involvement, diabetic neuropathy is, therefore, considered a microvascular disease. Current diabetic animal models simulate human diabetic neuropathy in terms of nerve conduction velocities, blood flow, and biochemical abnormalities but tend to have less severe microangiopathy. Therefore, we induced diabetic neuropathy in our DPN model rats by combining two well-established models of both diabetes and of ischemia-reperfusion [12].

Our results showed that the sciatic and tail nerve conduction velocity and sciatic nerve action potential amplitude of the DPN rats were significantly slower than those of normal rats (*p* < 0.01). However, following treatment with increasing doses of TTF, tail nerve conduction velocity of the DPN model rats increased in a dose-dependent manner. Interestingly, the improvement in sciatic nerve conduction velocity and action potential amplitude was most efficacious in the medium-dose group; however, the reason for this remains unclear and requires further investigation.

Diabetes and hyperglycemia also lead to increased expression of E-selectin, VCAM-1, and ICAM-1 by endothelial cells [26–28]. Intercellular adhesion molecule- (ICAM-) 1 is an immunoglobulin- (Ig-) like cell adhesion molecule expressed by several cell types including leukocytes and endothelial cells. ICAM-1 plays an important role in both innate and adaptive immune responses. It is involved in the transendothelial migration of leukocytes to sites of inflammation, as well as interactions between antigen presenting cells (APC) and T cells during immunological synapse formation. Increased ICAM-1 expression in the endothelial cell surface attracts monocytes, neutrophils, and T lymphocytes and endothelial cells, resulting in direct endothelial cell injury by proteases and oxygen free radical release [29]. Previous studies have found that blocking ICAM-1 can prevent leukocytosis as well as retinal vascular leakage [14].

Tumor necrosis factor-*α* (TNF-*α*) is a 25 kD molecular weight membrane protein mainly produced by macrophages and monocytes. The expression of circulating TNF-*α* is elevated in both streptozotocin-induced diabetic rats as well as diabetic patients [30]. Due to this association, this cytokine has potential to be a biomarker for DPN [31, 32]. Treatment of streptozotocin-induced diabetic rats with high-dose aspirin, the cyclooxygenase-2 inhibitor meloxicam, or etanercept, a competitive inhibitor of TNF-*α*, prevents increased ICAM-1 expression, leukostasis, capillary leakage, and upregulation of eNOS [14].

Myeloperoxidase (MPO), a neutrophil-specific reductase, is most abundantly expressed in the azurophilic granules of polymorphonuclear neutrophils (PMN). Analysis of MPO activity of nerve tissue may, therefore, reflect the extent of PMN extravasation from the microvasculature [33, 34]. Our results show that, compared with normal rats, ICAM-1 and TNF-*α* expression in sciatic nerve are significantly higher in DPN model rats. Additionally, increased MPO expression reflects the extent of PMN extravasation, and, indirectly, an increase in absolute number of neutrophils. This suggests that there is a series of inflammatory reactions* in vivo* caused by ischemia in the ischemia-reperfusion DPN model rats.

We also found that the plasma ICAM-1 and MPO expression levels in the DPN model rats are significantly higher than the control group (*p* < 0.01), further confirming the existence of ICAM-1-mediated inflammatory reaction* in vivo* of the DPN model rats. TTF treatment causes a significant dose-dependent decrease in both ICAM-1 expression and MPO and TNF-*α* expression (*p* < 0.01). These data show that the TTF can inhibit the plasma level of TNF-*α* and ICAM-1, thereby reducing the extent of the polymorphonuclear neutrophils tissue invasion from the microvasculature.

Previous studies have found that there are three major histopathological changes that affect the peripheral nerves in diabetic patients. First, thickening and proliferation of the endoneurium capillary wall and the stratum basale occur [35, 36]; second, capillary density and area decrease and become obstructed [35–37]; third, fiber loss is multifocal, suggesting an ischemic etiology [38, 39]. Of importance, similar histopathological changes were also present in the DPN model established in this study. Our results also showed sciatic capillary wall thickening, occlusion, and thrombosis in DPN model rats. Additionally, we observed that sciatic nerve fiber density decreased significantly, accompanied with uneven distribution, endoneurial edema, and axonal atrophy and deformity due to myelin extrusion. After TTF therapy, the occluded capillaries gradually returned to normal, and the number of abnormal nerve fibers and edema of the endoneurium decreased significantly.

Modern pharmacological studies have shown that the major components in TTF have significant antiplatelet aggregation and anti-inflammatory effects. For example, the total saponins of* Astragalus* (ASTs), the active constituent of* Astragalus*, can reduce whole blood viscosity and plasma viscosity, shorten erythrocyte electrophoresis time and erythrocytic specific volume [40], and have significant antithrombotic effects [41].* Astragalus* extract also has a significant protective effect on vascular endothelial injury by reducing the content of ICAM-1 and propylene glycol (MDA) and increasing superoxide dismutase (SOD) content. This protective effect is attributed to inhibition of inflammatory cell infiltration, reduction of inflammation, and scavenging oxygen free radicals [42, 43]. Ligustrazine (LTZ), the active constituent of* Ligusticum wallichii*, and total glucosides of* Paeonia* (TGP) both inhibit platelet aggregation and also improve hemorheology [44]. Radix Paeoniae Alba extract can inhibit the division of pUC18 DNA induced by phenol, clear peroxide, and hydroxyl radicals [45]. Curcumin, the effective constituent of* Curcuma*, can inhibit platelet aggregation and has a protective effect on ischemia-reperfusion injury [46].* Eupolyphaga* can effectively reduce blood viscosity and fibrinogen and inhibit thrombosis and platelet aggregation. Finally,* Eupolyphaga* water extract has an effect on antioxidation, serves as an anti-free radical, and protects vascular endothelial cells [47–49].

## 5. Conclusions

In the present study, we demonstrated that TTF treatment reduces the expression of ICAM-1, TNF-*α*, and MPO in the nerve tissue, which in turn reduces the blood viscosity and alleviates nerve ischemia, thereby improving peripheral nerve conduction velocity. In order to further understand the mechanism underlying the protective effect of TTF on DPN, future study is required to examine the effect of TTF on the signaling pathway upstream of ICAM-1, including levels oxidative stress and inflammatory cytokines.
